# Spontaneous resolution of foveal detachment in traction maculopathy in high myopia unrelated to posterior vitreous detachment

**DOI:** 10.1186/s12886-016-0195-3

**Published:** 2016-02-11

**Authors:** Tso-Ting Lai, Tzyy-Chang Ho, Chung-May Yang

**Affiliations:** Department of Ophthalmology, National Taiwan University Hospital, No. 7, Chun-Shan S. Rd., Taipei City, 100 Taiwan; College of Medicine, National Taiwan University, No.1 Jen-Ai Rd. Sec. 1, Taipei City, 100 Taiwan

**Keywords:** Foveal detachment, High myopia, Foveoschisis, Optical coherence tomography, Posterior vitreous detachment

## Abstract

**Background:**

Foveal detachment associated with foveoschisis usually takes a progressive course, and is associated with a poor visual outcome. The purpose of this study was to report the spontaneous resolution of foveal detachment in patients with myopic traction maculopathy without posterior vitreous detachment.

**Methods:**

A retrospective study involving eight cases of high myopia with foveoschisis and foveal detachment in which the subfoveal fluid had spontaneously resolved. The clinical characteristics and optical coherence tomography (OCT) findings were described.

**Results:**

All cases involved predominant schisis in the outer retina, with six showing internal limiting membrane detachment. The average central foveal thickness was 445.1 μm, and the average foveal detachment height was 271.5 μm. None of the cases involved traction of the vitreomacular interface or posterior vitreous detachment (PVD), either before or after the resolution of foveal detachment. In seven cases, the mean best-corrected visual acuity improved after foveal reattachment.

**Conclusions:**

Spontaneous reattachment not associated with PVD can occur in cases of high myopic traction maculopathy, especially in those without obvious vitreomacular traction.

## Background

Myopic foveoschisis is a complication of high myopia that can affect visual function. Studies have suggested that this condition usually takes a progressive course, with only a minority of cases remaining stable during follow-up [1–3]. Foveal detachment occurs in 34.5–72.0 % of patients with this condition, either at the time of diagnosis or during follow-up [1–3]; this is usually associated with a poor visual outcome [1]. However, a recent study involving 207 patients showed a much more stable clinical course, with the myopic foveoschisis progressing in only 11.6 % of cases during a follow-up period of more than 2 years [4]. Moreover, several investigations have suggested that surgical interventions, such as gas tamponade or vitrectomy, improve anatomical and functional outcomes in patients with this condition [1, 2, 5–7]. Nonetheless, complications such as macular hole formation have been reported [1, 5, 6], and functional outcomes may not parallel structural improvements [8, 9].

With regard to pathogenesis, foveoschisis is related to strong pre-retinal traction combined with axial length elongation [1, 3, 10–12]. In a previous report involving spontaneous resolution of macular detachment, a complete posterior vitreous detachment (PVD) occurred *before* the reattachment of the macula; for this reason, the investigators suggested that the PVD contributed to the reattachment [8]. Similarly, another recent study suggested that rupture of the internal limiting membrane (ILM) reduces traction and leads to resolution of schisis and foveal detachment [4]. In this study, which involved eight patients, we report the spontaneous resolution of foveal detachment in myopic tractional maculopathy that is unassociated with PVD or ILM rupture. In addition, we present the clinical course, as well as the findings from optical coherence tomography (OCT). Finally, various factors that may be associated with this spontaneous improvement are discussed.

## Methods

We retrospectively reviewed the cases of eight consecutive patients with myopic foveoschisis in whom a foveal detachment had spontaneously resolved; all patients had been diagnosed between January 2009 and December 2014. OCT images were traced backward to the patients’ original visits. All patients had been followed up by the Department of Ophthalmology at the National Taiwan University Hospital, and written informed consent had been obtained from all patients. Approval for this study was obtained from the National Taiwan University Hospital Research Ethics Committee, and it adhered to the tenets of the Declaration of Helsinki.

High myopia was defined as myopia of ≥ 6 diopters, and/or axial length ≥ 26 mm; spontaneous resolution of foveal detachment was defined as reattachment of the fovea without any surgical intervention (gas injection or vitrectomy). Clinical data were collected by retrospective chart review in all cases; these data included patient age, gender, auto-refraction, best-corrected visual acuity (BCVA), axial length (measured using the LENSTAR LS 900; Haag-Streit USA Inc., Mason, OH, USA), and findings from both slit-lamp and dilated fundus examinations. Findings from serial OCT images (spectral domain OCT, Cirrus™; HD-OCT, Carl Zeiss Meditec, Inc., Dublin, CA, USA; or RTVue™ RT100 version 3.5; Optovue Inc., Fremont, CA, USA) were recorded during the follow-up period. These included inner plexiform layer (IPL) retinoschisis [3], outer retinal retinoschisis, lamellar hole, ILM detachment, inner segment/outer segment (IS/OS) junction defect, and vitreoretinal interface traction. We carefully reviewed all the OCT images, which were obtained from a 5-line raster scan of the fovea, as well as from a macular cube scan of the entire macular area. Retinoschisis on the OCT images was classified according to the extent of outer schisis, as described by Shimada [4]: no macular retinoschisis (S0), extra-foveal retinoschisis (S1), fovea-only retinoschisis (S2), foveal and partial macular area retinoschisis (S3), entire macular area retinoschisis (S4). The height of the foveal detachment was defined as the largest distance, measured manually, from the surface of the retinal pigment epithelium (RPE) to the photoreceptor cell ellipsoid zone. The longest of these measurements in the series of OCT images was chosen as the foveal detachment height for each particular patient. The central foveal thickness (CFT) was measured at the same site, and was defined as the distance between the RPE and the inner retinal surface. Again, the longest of these measurements in the series of OCT images was chosen as the central foveal thickness [7]. The presence of PVD was determined by OCT examination, funduscopic findings, or clinical records of previous ocular surgery. In patients who had developed foveal detachment during follow-up after an initial presentation of only foveoschisis (patient Nos. 1–4 in Table 1), the duration of foveal detachment was defined as the interval between the first date on which OCT had shown foveal detachment and the first date on which it had shown complete resolution of foveal detachment.

**Table 1:** Clinical features of patients with myopic foveoschisis combined with a foveal detachment that spontaneously resolved

Patient no.	Age (years)	Gender	RE (Diopter)	Axial length (mm)	Chorioretinal atrophy	Clinical course	Serial BCVA^a^ (logMAR)	Duration of FD (months)
1	61–65	Woman	IOL	28.78	D + P	FS → FS + FD → FS	0.8 / 0.8 / 0.8	31
2	51–55	Woman	−18.37	30.47	D	FS → FS + FD → FS	0.7 / 1.0 / 0.8	27
3	41–45	Woman	−17.0	31.05	D + P	FS → FS + FD → FS	0.5 / 1.3 / 1.0	24
4	41–45	Man	−13.25	29.85	D	FS → FS + FD → FS	0.1 / 0.3 / 0.05	6
5	66–70	Man	−6.37	28.32	D	FS + FD → no FS/FD^b^	NA / 2.0 / 1.5	NA
6	41–45	Woman	−13.0	28.36	D	FS + FD → FS	NA / 0.4 / 0.1	NA
7	51–55	Woman	−11.75	NA	D	FS + FD → FS	NA / 2.0 / 1.0	NA
8	66–70	Woman	IOL	30.36	D	FS + FD → FS	NA / 0.5 / 0.3	NA
Mean ± SD	54.6 ± 10.0		−13.3 ± 3.9	29.6 ± 1.0				22.0 ± 9.6

*BCVA* best-corrected visual acuity, *D* diffuse atrophy, *FD* foveal detachment, *FS* foveoschisis, *IOL* intraocular lens, *NA* not available, *P* patchy atrophy, *RE* refractive error, *RPE* retina pigment epithelium, *SD* standard deviation

^a^The serial BCVA was recorded in the following order: with FS but before FD–during FS and FD–after FD had resolved

^b^In patient No. 5, the FS and FD both resolved completely during follow-up

### Statistical analysis

The change in BCVA (measured in logarithm of the minimum angle of resolution [logMAR]) after resolution of the foveal detachment was analyzed using the student’s t-test for paired samples; *p*-values < 0.05 were considered statistically significant.

## Results

The clinical characteristics of the patients are listed in Table 1. The mean age was 54.6 ± 10.0 years (range = 44–68 years). The average spherical equivalent measured was −13.3 ± 3.9 diopters (range = −18.37−6.37) and the average axial length measured was 29.6 ± 1.0 mm (range = 28.32–31.05). The refractive error before cataract surgery was not available for patient Nos. 1 and 8. Diffuse chorioretinal atrophy was noted in all cases, while patchy atrophy over the macular area was noted in patient Nos. 1 and 3. Four patients had an initial presentation of foveoschisis and foveal detachment; the other four developed foveal detachment during the follow-up period. The mean duration of foveal detachment in the latter four cases was 22.0 ± 9.6 months (range = 6–31 months). In seven of the eight patients, BCVA improved after the subretinal fluid had resolved, and mean BCVA was significantly better after foveal detachment had resolved compared with when the fovea was detached (*p* < 0.05).

**Fig. 1:**
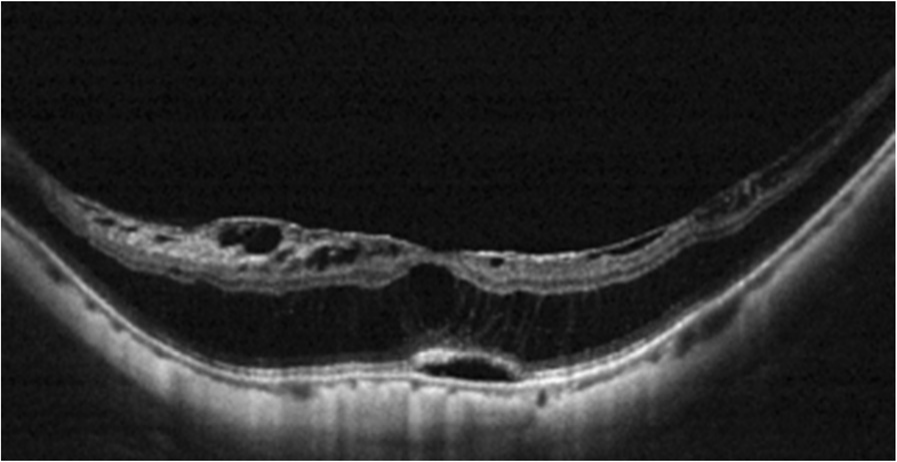
Optical coherence tomography (OCT) image from patient No. 2. Representative OCT image from patient No. 2 showing typical findings: ILM detachment, retinoschisis, disruption of the IS/OS junction, and foveal detachment. The schisis was more prominent in the outer retina, and no vitreoretinal interface traction or outer laminar break was seen

The OCT findings are summarized in Table 2, and Fig. 1 shows a typical example of such findings. All patients had S4 retinoschisis, except for patient No. 5, who had foveal and partial macular area retinoschisis, which was therefore classified as S3. None of the patients had visible vitreoretinal interface traction (epiretinal membrane or vitreomacular traction). In all patients, except for No. 4, the IS/OS junction was disrupted after the retina had reattached. Patient 6 presented with retinoschisis, foveal detachment, and an inner lamellar hole at the initial visit. In addition, the lamellar hole continued to increase in size for as long as the schisis persisted, despite the resolution of subretinal fluid. An *outer* lamellar hole was noted at initial presentation in patient No. 8, who also had foveal detachment and schisis. All cases of retinoschisis mainly involved the outer retina; ILM detachment was noted in six cases. After the foveal detachment had resolved, the schisis remained the same in four cases, became less prominent in three cases, and completely resolved in one case. The average height of foveal detachment was 271.5 ± 68.2 μm (range = 191–396 μm). The mean CFT was 445.1 ± 79.6 μm (range = 330–550 μm). Two representative cases are presented here:

**Table 2:** Optical coherence tomography findings from patients with myopic foveoschisis and foveal detachment that spontaneously resolved

Patient no.	Classification^a^	ERM/VMT	ILM detachment	Retinoschisis	LH	Persisted schisis	IS/OS junction disruption	FD (μm)	CFT (μm)
1	S4	Removed^b^	+	Outer	-	+	+	272	493
2	S4	-	+	Inner & outer^c^	-	+	+	230	482
3	S4	-	+	Outer	-	Partial^d^	+	230	341
4	S4	-	+	Outer	-	+	-	191	499
5	S3	-	-	Outer	-	-	+	371	499
6	S4	-	+	Inner & outer^c^	+^e^	+	+	242	367
7	S4	-	-	Outer	-	Partial^d^	+	240	330
8	S4	-	+	Outer	+^f^	Partial^d^	+	396	550

*CFT* central foveal thickness, *ERM* epiretinal membrane, *FD* the height of foveal detachment, *ILM* internal limiting membrane, *IS/OS* inner segment/outer segment, *LH* lamellar hole, *VMT* vitreomacular traction

+ = present; − = absent

^a^The classification of retinoschisis was based on the extent of outer retinoschisis described by Shimada [4], where schisis involving the fovea and partial macula was classified as S3, and schisis involving the entire macula area was classified as S4

^b^In patient No. 1, the ERM was removed via vitrectomy without ILM peeling one month before the development of foveal detachment

^c^In patient Nos. 2 and 6, the retinoschisis involved both the inner (inner plexiform layer) and outer retina

^d^In patient Nos. 3, 7, and 8, the retinoschisis had decreased, with some residual schisis in the outer retina

^e^In patient No. 6, an inner lamellar hole was noted at initial presentation, with enlargement during follow-up

^f^In patient No. 8, an outer lamellar hole was noted at initial presentation

### Report of cases

#### Patient No. 5

**Fig. 2:**
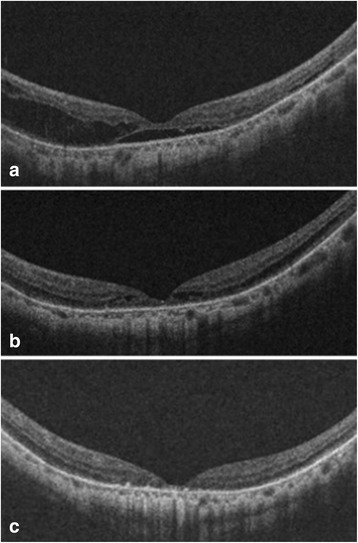
Serial optical coherence tomography (OCT) images from patient No. 5. Serial OCT images from a 68-year-old man (patient No. 5) demonstrate spontaneous resolution of foveoschisis and foveal detachment. **a** At presentation, OCT showed foveoschisis of the outer retina combined with foveal detachment. **b** One year later, the subretinal fluid had resolved spontaneously, and the severity of schisis had decreased. **c** Four months after the detachment had resolved, OCT revealed complete resolution of foveoschisis, as well as some tissue loss in the outer retina and disruption of the IS/OS junction

A 68-year-old man without any specific underlying disease was referred to our clinic and received scleral buckling to treat retinal detachment in the right eye. During the preoperative evaluation, poor vision in the left eye was noted, with BCVA being 2.0 logMAR. Fundoscopy revealed a typical myopic fundus with diffuse atrophy. OCT showed foveal detachment with foveoschisis in the left eye (Fig. 2). The schisis mainly occurred in the outer retina, without notable ILM detachment or lamellar hole formation. Furthermore, there was no macular hole or visible premacular traction. The axial lengths were 27.40 mm and 28.32 mm in the right and left eyes, respectively. The subretinal fluid, as well as the schisis in the left eye, gradually decreased during follow-up, and the retina had reattached one year later. Specifically, the schisis had resolved completely four months after the reattachment of the fovea—OCT revealed an attached retina with a disruption of the IS/OS junction. The patient’s BCVA in the left eye was 1.5 logMAR at the last visit, 23 months after the initial presentation.

#### Patient No. 6

**Fig. 3:**
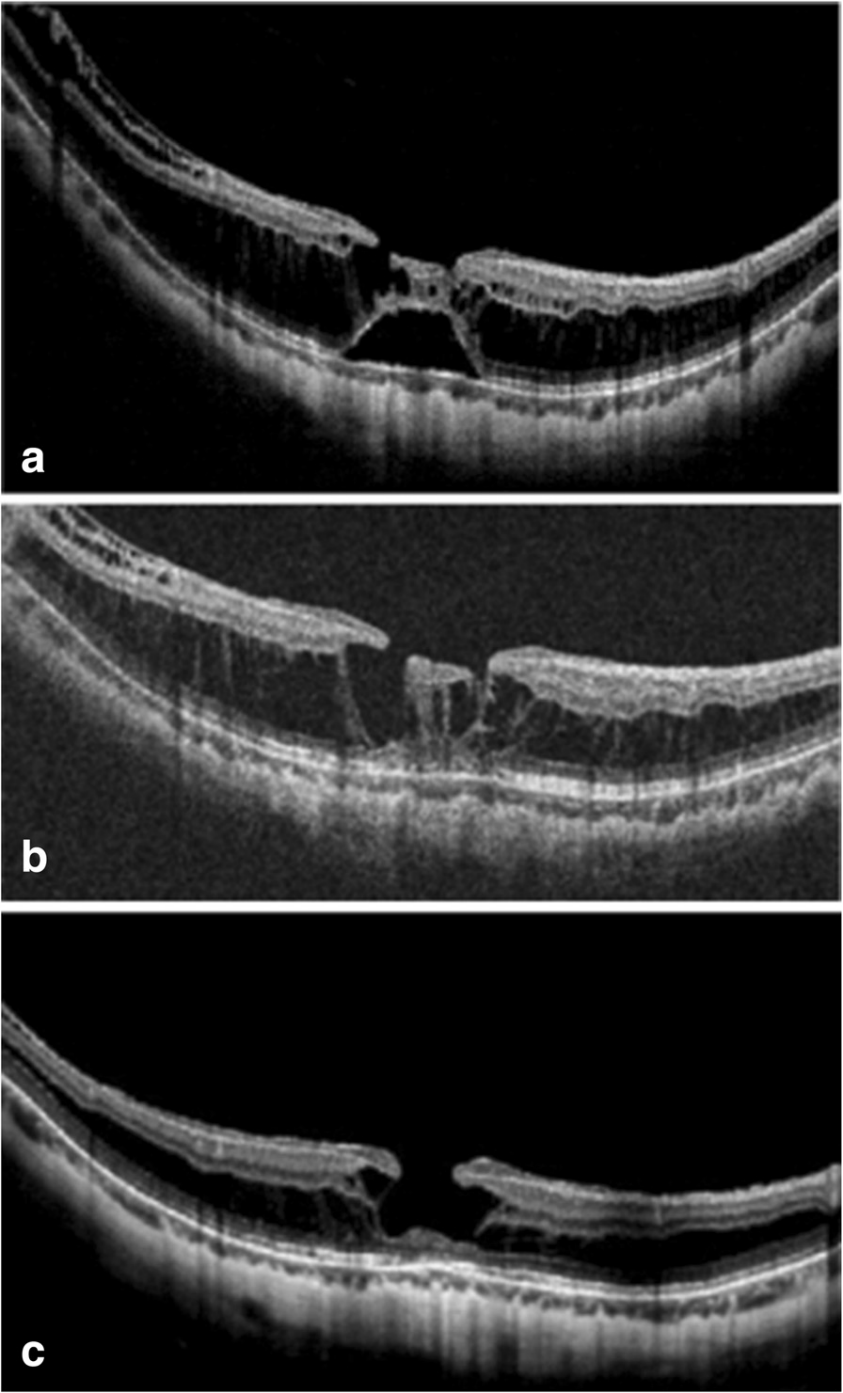
Serial optical coherence tomography (OCT) images from patient No. 6. The serial OCT images from a 44-year-old woman with high myopia (patient No. 6) show spontaneously resolved foveal detachment and enlarged inner lamellar hole. **a** The OCT at initial presentation showed retinoschisis, mainly over the outer retina, and ILM detachment. An inner lamellar hole was also present. No vitreomacular interface traction was noted. **b** The retina had reattached 6 months later, without operation, with disruption to the IS/OS junction visible on the same image. The schisis, ILM detachment, and inner lamellar hole persisted. **c** After 44 months, the macula remained attached, while the inner lamellar hole had enlarged without the formation of a macular hole. The retinoschisis over the outer retina was still visible, as was the disruption to the IS/OS junction

A 44-year-old woman with high myopia presented with a history of vitrectomy to treat retinal detachment in the right eye. She complained of decreased vision in the left eye, which had persisted for one month prior to her initial visit to our hospital. Fundoscopy revealed a tessellated fundus with diffuse atrophy in the left eye; OCT showed foveoschisis with foveal detachment, as well as an inner lamellar hole (Fig. 3). Moreover, ILM detachment was also noted on the OCT image. The patient’s BCVA was 0.4 logMAR, and her axial length was 28.36 mm in the left eye. Six months later, the retina had reattached without treatment, and the patient’s BCVA had improved to 0.1 logMAR. Although foveoschisis persisted, and the lamellar hole continued to enlarge during the subsequent two years, no macular hole formed. The final BCVA in the left eye was 0.4 logMAR, with cataract progression.

## Discussion

The evolution of foveoschisis in high myopia can be diverse. For instance, it is generally believed that the condition takes a progressive course in most cases; however, in a recent study involving the most patients with myopic retinoschisis reported in any investigation to date, the disease had progressed in only 24 (11.6 %) of 207 eyes during follow-up [4], with retinal detachment developing in 7 of those 24. The same researchers reported that the schisis had spontaneously improved in 8 of the 207 eyes, and it had completely resolved in 6 of those. In this study, we analyzed eight cases of foveoschisis in which foveal detachment spontaneously resolved during follow-up without obvious changes in the vitreoretinal relationship. These clinical courses imply that myopic foveoschisis, and the foveal detachment associated with it, has a more complex etiology.

The pathogenesis of foveoschisis is related to strong traction on the retina, which is in turn caused by rigidity of the ILM, posterior vitreous, epiretinal membrane, and retinal vessels [3, 10]. Increases in the axial length, which are associated with pathological myopia, also play a role in generating tractional force from the tissues listed above. Previous studies have associated various factors with the development of foveal detachment and foveoschisis: axial length > 31 mm, macular chorioretinal atrophy, and vitreoretinal interface factors [12], as well as ILM detachment, and IPL retinoschisis [3]. However, the average axial length measured in our study was 29.6 mm, and only one patient had an axial length over 31 mm. In addition, none of our patients had any traction in the vitreomacular interface that was visible by either fundoscopy or OCT. The relatively short axial length, as well as the absence of visible vitreomacular interface factors, indicates less severe traction on the retina, which may contribute to the spontaneous resolution of foveal detachment in our study.

Using OCT, Fujimoto et al. showed a higher incidence of ILM detachment and IPL retinoschisis in foveoschisis patients with foveal detachment than in those without detachment [3]. These findings may suggest that a stronger inward traction is generated at the vitreous membrane, as well as at the rigid ILM, and that this is transmitted to the outer retina through the columnar structure in the layers of the retinoschisis. Likewise, Shimada et al. reported two cases of ILM disruption prior to spontaneous resolution of retinoschisis, and assumed that the disruption of the already detached ILM releases the severe traction [4]. In our study, the retinoschises were mainly confined to the outer retina, and ILM detachment was noted in six eyes (75 %). No disruption of any detached ILM was noted in our study, at least in the scanning area. The lack of IPL retinoschisis in six of our eight cases may indicate a less severe traction, and this may serve as a predictive factor for spontaneous resolution of the subretinal fluid.

The lower foveal detachment height and shorter CFT provide further evidence for less severe traction in our patients than in those from a previous study who had foveoschisis and foveal detachment that showed a progressive course and required surgery [7]. In our previous study on the treatment of foveoschisis, the average heights of foveal detachment (576.9 ± 378.6 μm) and CFT (762.8 ± 314.7 μm) were significantly greater than in this study (*p* < 0.05). Thus, we would suggest a quantitative measurement of these two parameters before considering surgical treatment.

A previous study from Shimada et al. found that the formation of an outer lamellar hole predisposes a patient to foveal detachment in myopic retinoschisis [13]. In none of our patients did any lamellar hole occur during the follow-up period, except for patient No. 8, who had an outer lamellar hole at the initial presentation. Foveal detachment developed *during* the follow-up period in four cases in our study, and the OCT images from these cases showed no evidence of outer lamellar hole formation before the retinal detachment. However, it is uncertain whether this really indicates a favorable disease process; it may simply have been due to inadequate scanning, because only a small cubic region of the macular area was examined. One eye in our series had an inner lamellar hole, which enlarged after the subretinal fluid resolved. The enlarged opening of the hole in this eye may have released some of the traction; in fact, loss of the columnar structure of the retina, caused by the hole itself, may actually have decreased transmission of the inward traction. Both factors may have played a role in the resolution of the foveal detachment.

In one previous report in which macular detachment spontaneously resolved, complete PVD occurred before the reattachment of macula; the investigators therefore postulated PVD as a contributing factor to the reattachment [8]. Indeed, PVD occurred in four of the eight eyes with decreased or resolved retinoschisis that were reported by Shimada et al. [4], and in three of the four eyes reported by Hirota et al. in which traction maculopathy had resolved [9]. Nonetheless, no such association was observed in our study. One of our patients had received pars plana vitrectomy (PPV) long before the macular detachment, and PVD was induced during that operation; in none of the others was PVD found, whether by OCT or fundoscopy. This was true both before and after the foveal detachment had resolved. Although there was no obvious evidence of vitreomacular membrane or PVD in our cases, even after all the OCT images had been carefully reviewed, it remains possible that posterior hyaloid detachment was not detected by OCT before the resolution of foveal detachment [12]. Nonetheless, as our cases were different from those of previous reports, we believe a different mechanism may have been involved in the formation of foveal detachment, and factors other than PVD may have been responsible for the resolution of macular detachment in the eyes without detectable vitreomacular interface tractions.

In previous studies, PPV with or without ILM peeling has been used to treat foveal detachment and foveoschisis [1, 2, 5, 6]; the procedure is generally associated with a positive visual outcome. A less invasive procedure using the gas tamponade has also been proposed to treat myopic foveoschisis with foveal detachment, and also results in favorable outcomes [7]. However, macular hole formation is not an uncommon complication after vitrectomy to treat myopic foveoschisis [1, 5, 6], especially in those with IS/OS junction defect [6]. In our study, although seven of the eight patients had IS/OS junction defect, none developed macular hole after their foveal detachment had resolved spontaneously.

Subretinal fluid was the main cause of visual impairment among our patients; visual acuity improved significantly after the retina reattached, even in those with longstanding subretinal fluid and IS/OS junction defect. Nonetheless, the final visual acuity was poor in most cases, because of the duration for which the subretinal fluid and the disruption to the IS/OS junction had persisted.

The present study was limited by its small number of patients. Furthermore, as it was retrospective in nature, it lacked a well-designed control group. Nonetheless, the investigation constituted a longitudinal follow-up, over a relatively long period, of patients with foveoschisis and foveal detachment. Furthermore, our report presents the common OCT findings in such cases. We found that foveal detachment can spontaneously resolve through previously unreported mechanisms; this should be taken into consideration before surgery is planned, especially in eyes with a relatively short axial length, a predominant outer segment retinoschisis, or no obvious vitreomacular interface traction. Further studies are needed, using a greater number of cases and well controlled approaches, to determine the exact predictive factors for this different clinical course.

## Conclusions

Spontaneous reattachment of foveal detachment and resolution of foveoschisis can occur in cases with high myopic traction maculopathy without evidence of PVD, especially in those without obvious vitreomacular tractions. This possibility should be taken into consideration before surgery is planned in such cases.

## Abbreviations

BCVA: best-corrected visual acuity
CFT: central foveal thickness
ILM: internal limiting membrane
IPL: inner plexiform layer
IS/OS: inner segment/outer segment
OCT: optical coherence tomography
PPV: pars plana vitrectomy
PVD: posterior vitreous detachment
